# Rectal cancer surgery in older people does not increase postoperative complications - a retrospective analysis

**DOI:** 10.1186/1477-7819-12-355

**Published:** 2014-11-23

**Authors:** Jagdeep Singh, Anton Stift, Sarah Brus, Katharina Kosma, Martina Mittlböck, Stefan Riss

**Affiliations:** Department of Surgery, Medical University of Vienna, Währinger Gürtel 18-20, A-1090 Vienna, Austria; Center for Medical Statistics, Informatics and Intelligent Systems, Medical University of Vienna, Vienna, Austria

**Keywords:** Rectal cancer, Rectal surgery, Older patients, Risk factors, Postoperative complications

## Abstract

**Background:**

Rectal cancer surgery in the older population remains a highly controversial topic. The present study was designed to assess whether older patients had an increased risk for postoperative complications after rectal resection for malignancies.

**Methods:**

Consecutive patients (n =627), who underwent rectal cancer resection at a single institution, were included in the study and analyzed retrospectively. Short-term complications were compared between patients ≥80 years (n =55) and <80 years (n =572). Additionally, predictive factors for postoperative complications were analyzed.

**Results:**

The older aged group showed a significantly higher rate of co-morbidities compared to controls, in terms of cardiovascular and pulmonary diseases (*P* =0.002, *P* =0.006). In older patients, a Hartmann’s procedure and transanal endoscopic microsurgery (TEM) were performed most frequently (*P* <0.0001).

The overall complication rate was 39% (n =244) (medical: n =59 (9%), surgical: n =185 (30%)), including 24 (44%) complications in the older aged group (medical: n =6 (11%), surgical: n =18 (33%)). Notably, the incidence of surgical and medical complications showed no significant difference between patients and controls (*P* =0.58, *P* =0.69).

Neurological and cardiovascular disorders were associated with an increased risk for a eventful postoperative course in the older aged group (*P* =0.03, *P* =0.04).

**Conclusions:**

Rectal cancer resection can be performed safely in selected older patients. Age itself should not be considered as a risk factor for postoperative complications.

## Background

Colorectal cancer is the third most common malignancy worldwide with an increasing incidence over the last decade, especially in the older population [1]. In the light of increasing life expectancy, the medical health system will be confronted with a significantly growing number of older patients in the upcoming years. This development will not only change our daily clinical routine but also challenge the decision-making in the treatment of malignancies in this generation. The careful assessment of the patients will become an essential and central issue and choosing the appropriate treatment for each patient will rely on a multidisciplinary process.

Older people often have a significant number of co-morbidities, thus clinicians tend to offer less aggressive oncological treatment, with the potential consequences of early disease recurrence and reduced overall survival.

In the literature, the optimal treatment of rectal cancer in older aged patients remains controversial [2]. In addition, the interpretation of current data needs to be done with caution as most studies included both colonic and rectal cancer, although treatment strategies and morbidity rates vary significantly. Secondly, the definition of the older aged population is inconsistent with an age range of between 65 and 85 years.

Several studies have described that older age alone is not a risk factor for postoperative complications for patients with rectal cancer and therefore does not increase the mortality rate [3–7]. In contrast, other studies found out that increased age correlated with a higher postoperative mortality rate and lower overall survival [8–10]. Notably, the occurrence of co-morbidities influenced the mortality rate significantly [3, 11, 12].

The present study was designed to evaluate whether older patients (>80 years) have an increased risk for postoperative complications after rectal cancer surgery. In addition, we aimed to define parameters to predict an eventful postoperative course in this group of patients.

## Methods

Between 1997 and 2010, 627 patients (female: n =248 (40%), male: n =379 (60%), aged 18 to 92 years), who underwent rectal cancer resection at the Department of Surgery, Medical University of Vienna, were included in the study and analyzed retrospectively.

Rectal cancer surgery was divided into intersphincteric or complete rectal resection with coloanal anastomosis and low anterior resection with colorectal anastomosis. A transanal endoscopic microsurgery (TEM), a Hartmann’s procedure or an abdominoperineal resection was conducted in selected cases.

The investigation was approved by the local ethics committee. Data were collected from the institutional colorectal database and individual chart reviews respectively.

Postoperative complications were defined as complications during the first 30 days after surgery and divided into surgical and medical. In addition, postoperative complications were graded according to the Clavien-Dindo classification [13].

Short-term complication rates were compared between patients ≥80 years and controls (<80 years).

In addition, 110 patients, 55 in each group, were selected for a matched pair analysis. The control group was matched by sex, type of neoadjuvant therapy and type of operation. Hereby, we aimed to achieve more accurate data, as radio/chemotherapy and the type of surgical procedure may also have an impact on the outcome.

Predictive factors for postoperative complications in older patients were analyzed: age, gender, smoking status, co-morbidities, tumor localization, tumor staging, UICC-criteria (Union Internationale Contre le Cancer), preoperative therapy and type of operation.

### Statistical analyses

The chi-square test, the exact chi-square test and the Fisher’s exact test were used to assess associations between categorical variables. In case of ordinal variables a trend version of the chi-square test was used. For matched data analyses the McNemar test was used for binary variables and a test of symmetry for nominal data. All *P*-values are two-sided and *P* ≤0.05 was considered significant. All calculations were performed with the statistical analysis software SAS (SAS Institute Inc., Version 9.3, Cary, NC, USA).

## Results

### Patients (≥80 years) and controls (<80 years)

Demographic data of patients ≥80 years and controls (<80 years) are outlined in Table 1.

**Table 1:** **Demographic data of patients (≥80 years) and controls (<80 years)**

	Group 1	Group 2	***P***-value
	Age <80 years	Age ≥80 years	
	n =572	n =55	
Gender			0.071
Female	220 (38)	28 (51)	
Male	352 (62)	27 (49)	
Co-morbidities			
Cardiovascular diseases	250 (44)	36 (65)	0.002
Pulmonary diseases	63 (11)	13 (24)	0.006
Neurological diseases	34 (6)	6 (11)	0.149
Diabetes mellitus	84 (15)	9 (16)	0.738
Tumor localizations			0.432
Upper rectum (12 to 16 cm)	107 (19)	7 (13)	
Middle rectum (6 to 12 cm)	220 (39)	25 (46)	
Lower rectum (≤6 cm)	241 (42)	22 (41)	
UICC-criteria			0.109
I	174 (31)	21 (41)	
II	117 (21)	10 (20)	
III	156 (28)	14 (27)	
IV	109 (20)	6 (12)	
Operative access			0.0004
Open	511 (90)	44 (80)	
Laparoscopic	44 (7)	3 (5)	
Transanal	15 (3)	8 (15)	
Operation techniques			**<** 0.0001
Low anterior resection	374 (65)	28 (51)	
Rectum extirpation	65 (11)	6 (11)	
Intersphincteric resection	42 (7)	1 (2)	
Transanal endoscopic microsurgery	14 (3)	8 (14)	
Hartmann’s procedure	15 (3)	7 (13)	
Complete rectal resection	48 (8)	4 (7)	
Others	14 (3)	1 (2)	
Stomas			0.024
Colostoma	121 (21)	18 (33)	
Ileostoma	259 (45)	15 (27)	
No stoma	190 (33)	22 (40)	
Preoperative therapies			0.005
No therapy	253 (44)	37 (67)	
Chemo or radio/chemotherapy	125 (22)	7 (13)	
Radiotherapy	194 (44)	11 (20)	

Values are given as numbers and absolute frequencies (%).

The older aged group (n =55 (9%)) showed a significantly higher rate of co-morbidities compared to the control group. Especially, cardiovascular disease and pulmonary disease were observed more frequently in patients aged above 80 years (*P* =0.002 and *P* =0.006). The most common pulmonary disease was chronic obstructive pulmonary disease (COPD) (n =6 (11%)). Furthermore, 319 (56%) patients in the control group received neoadjuvant chemotherapy or radiotherapy compared to 18 (33%) of older patients (*P* =0.005).

Lower anterior resection was the most common operation in both groups. Notably, the older aged group received more TEMs and Hartmann’s procedures (n =8 (14%) and n =7 (13%)) but fewer intersphincteric resections (n =1 (2%)) and low anterior resections (n =28 (51%)) compared to the control group (n =14 (3%), n =15 (3%), n =42 (7%) and n =374 (65%)) (*P* <0.0001).

A total of 244 complications were observed in the present series. We found 24 (10%) complications in the older aged group (surgical: n =18 (33%) and medical: n =6 (11%)) and 220 (38%) complications in the control group (surgical: n =167 (29%) and medical: n =53 (9%)). Most common surgical and medical complications were wound infections (older age: n =6 (11%), control: n =45 (8%)), paralytic ileus (older age: n =2 (4%), control: n =33 (6%)), anastomotic leaks (older age: n =2 (4%), control: n =26 (5%)), urological complications (older age: n =4 (7%), control: n =26 (5%)), cardiovascular complications (older age: n =2 (4%), control: n =17 (3%)) and fever (older age: n =0, control: n =16 (3%)). They are further outlined in Table 2. Notably, no significant differences were detected between both groups (*P* =0.58, *P* =0.69).

**Table 2:** **Surgical and medical complications between patients (≥80 years) and controls (<80 years)**

	Group 1	Group 2	***P-***value
	Age <80 years	Age ≥80 years	
	n (%)	n (%)	
Surgical complications	167 (29)	18 (33)	0.583
Anastomotic leakage	26 (5)	2 (4)	
Wound infection	45 (8)	6 (11)	
Bleeding	6 (1)	0	
Paralytic ileus	33 (6)	2 (4)	
Mechanical obstruction	4 (1)	0	
Blood transfusion	7 (1)	1 (2)	
Intraabdominal abscess	6 (1)	0	
Urological complications	26 (5)	4 (7)	
Others	14 (3)	3 (6)	
Medical complications	53 (9)	6 (11)	0.690
Fever	16 (3)	0	
Thrombosis	3 (1)	0	
Pulmonary embolism	5 (1)	0	
Cardiovascular complications	17 (3)	2 (4)	
Pneumonia	2 (0)	1 (2)	
Others	10 (2)	3 (6)	

Values are given as numbers and absolute frequencies (%).

Additionally, no difference was observed between the older population and controls in regard to the Clavien-Dindo classification: in the older aged group, 6 (11%) patients were classified as grade I, 8 (15%) as grade II, 2 (4%) as grade IIIa and 6 (11%) as grade IIIb (*P* =0.35). None of the older patients died in the perioperative period.

Regarding the control group, 63 (11%) patients were classified as grade I, 55 (10%) as grade II, 14 (3%) as grade IIIa and 49 (9%) as grade IIIb. Six (1%) patients were classified as grade IV.

### Matched pair analysis

Apart from the matching criteria (type of neoadjuvant therapy, type of operation and sex), the height of tumor, tumor stage and the number of stoma creations were equally distributed between both groups. However, in the older aged group 13 (24%) patients presented with pulmonary diseases compared to 2 (4%) patients in the control group (*P* =0.005).

The overall complication rate in the older aged group was 40% (n =22) and 31% (n =17) in the control group (*P* =0.34). In addition, no difference was found between the older aged and control group in regard to surgical (n =18 (33%) and n =12 (22%)) and medical complications (n =6 (11%) and n =6 (11%)) (*P* =0.18 and *P* =1.0). According to the Clavien-Dindo classification no significant difference was observed either (*P* =0.95) (Table 3).

**Table 3:** **Clavien-Dindo classification of patients and matched controls**

	Match group 1	Match group 2	***P***-value
	Age <80 years	Age ≥80 years	
	n (%)	n (%)	
Patients without complications	39 (71)	33 (60)	
Clavien-Dindo grade			0.953
I	4 (7)	6 (11)	
II	5 (9)	8 (15)	
IIIa	1 (2)	2 (4)	
IIIb	5 (9)	6 (11)	
IV to V	1 (2)	0	

Values are given as numbers and absolute frequencies (%).

### Risk factors for postoperative complications in older patients

Neurological and cardiovascular diseases correlated with an increased risk for a postoperative eventful course within 30 days post-surgery. Five out of 6 patients with a neurological disease (83%) had postoperative complications within 30 days, compared to only 35% of the patients without a neurological disease (17 of 49 patients; *P* =0.033). Half of the patients with a cardiovascular disease (18 of 36), but only 21% of the patients without a cardiovascular disease (4 of 19 patients; *P* =0.037), developed postoperative complications. The detailed list of risk factors is shown in Table 4.

**Table 4:** **Risk factors for postoperative complications**

	Complications	Complications	***P***-value
	No	Yes	
Gender			0.920
Female	17 (61)	11 (39)	
Male	16 (59)	11 (41)	
UICC-criteria			0.114
I	16 (76)	5 (24)	
II	5 (50)	5 (50)	
III	7 (50)	7 (50)	
IV	3 (50)	3 (50)	
Preoperative therapy			0.591
No therapy	24 (65)	13 (35)	
Chemo or radio/chemotherapy	4 (57)	3 (43)	
Radiotherapy	5 (45	6 (55)	
Tumor localization			0.839
Upper rectum (12 to 16 cm)	4 (57)	3 (43)	
Middle rectum (6 to 12 cm)	15 (60)	10 (40)	
Lower rectum (≤6 cm)	14 (63)	8 (36)	
Operative access			0.024
Open	24 (55)	20 (45)	
Laparoscopic	1 (33)	2 (67)	
Transanal	8 (100)	0	
Operation technique			0.126
Low anterior resection	14 (50)	14 (50)	
Rectum extirpation	3 (50)	3 (50)	
Intersphincteric resection	1 (100)	0	
Transanal endoscopic microsurgery	8 (100)	0	
Hartmann’s procedure	3 (43)	4 (57)	
Complete rectal resection	3 (75)	1 (25)	
Others	1 (100)	0	
Anastomotic technique			0.354
No anastomosis	12 (63)	7 (37)	
Staple	15 (52)	14 (48)	
Manual	5 (83)	1 (17)	
Anastomotic form			0.500
No anastomosis	12 (63)	7 (37)	
J-pouch	0	1 (100)	
End-to-side anastomosis	1 (33)	2 (67)	
End-to-end anastomosis	20 (63)	12 (37)	
Stomas			0.645
Colostoma	10 (56)	8 (44)	
Ileostoma	8 (53)	7 (47)	
None	15 (68)	7 (32)	
Accessory surgery			0.253
None	23 (66)	12 (34)	
Accessory surgery	10 (50)	10 (50)	
Neurological diseases			0.033
No	32 (65)	17 (35)	
Yes	1 (17)	5 (83)	
Cardiovascular diseases			0.037
No	15 (79)	4 (21)	
Yes	18 (50)	18 (50)	
Diabetes mellitus			0.289
No	26 (57)	20 (43)	
Yes	7 (78)	2 (22)	
Pulmonary diseases			0.244
No	27 (64)	15 (36)	
Yes	6 (46)	7 (54)	
Smoker			0.760
No	26 (60)	17 (40)	
Yes	1 (33)	2 (67)	
No longer	6 (67)	3 (33)	

Values are given as numbers and absolute frequencies (%).

In addition, the type of anastomotic configurations had a significant impact on postoperative medical complications (*P* =0.048). However, a J-pouch was performed in only one patient, who afterwards developed a medical complication.

## Discussion

In the present analysis we could clearly demonstrate that rectal cancer resections can be conducted safely in selected older patients without an increased number of postoperative complications.

Surgery remains the only curative treatment for patients with rectal cancer [14]. However, no clear guidelines for a standardized management in older patients with colorectal cancer exist so far. Most available studies included older patient cohorts aged between 65 and 75 years, with only a paucity of studies investigating the outcome after rectal resections in patients over 80 years [3, 4, 10, 11, 15, 16].

Previous studies showed that colorectal surgery did not impair postoperative outcomes in older patients [3, 11, 15]. However, most of these studies comprised patients with both colon and rectal cancer. We analyzed patients with rectal cancer only and measured comparable numbers of medical and surgical complications. In 2006, Law *et al*. analyzed postoperative events in patients over 75 years compared with younger patients undergoing rectal surgery. Altogether, 37% of the patients in the older aged group had complications and 30% of the younger patients, leading to the conclusion that curative resection for rectal cancer could be performed safely in both age groups [11]. Vironen *et al*. assessed patients older than 75 years and found similar results in terms of complications after surgery [4].

Shahir *et al*. investigated treatment-related complications and overall survival in patients from the age of 60 years. The authors reported complication rates of 65% in patients >70 years and 51% in patients between 60 and 69 years. The older aged group was found to have more cardiac complications (8 versus 2%) and pneumonia (12 versus 7%) compared to younger controls. Furthermore, patients, who underwent surgery and received radiotherapy, had a significantly higher risk for postoperative complications than those undergoing surgery alone. Notably, in their series, the occurrence of complications was associated with an increase in the overall mortality rate [16]. In our series, none of the patients, who developed postoperative complications died during the observational period.

Few studies described older age as an independent risk factor for complications [17, 18]. We analyzed predictive factors for a postoperative eventful course in older patients too and found only cardiovascular and neurological diseases to be correlated with a higher risk for postoperative short-term complications. Law *et al*. obtained similar results, showing that cardiovascular and neurological diseases occurred more frequently in the older patients. Turrentine *et al*. investigated the morbidity and mortality rates and risk factors in older patients undergoing major operations [18]. Hypertension and dyspnea were significantly associated with an eventful postoperative course.

It is worth mentioning that a higher rate of Hartmann’s procedures was performed in the older aged group. Similar results were shown by Jung *et al*., where a total of 17% of the older patients received a Hartmann’s procedure in contrast to 5% of the younger patients [10]. Older patients who are fit for surgery often complain about a reduced anorectal function. In those patients a primary low anastomosis might cause postoperative fecal incontinence and subsequently deteriorate quality of life; thus, a definitive stoma might be a better option in the long term. Additionally, a Hartmann’s procedure has a shorter operative time and avoids possible anastomotic leakages with potential further interventions [19, 20].

The benefits of neoadjuvant radio/chemotherapy in rectal cancer treatment are well documented in the literature [21, 22]. However, the Stockholm I and II trials have shown the distinct negative effects of neoadjuvant radiotherapy in older patients (<80 years) [23–25]. The incidence of venous thromboembolism, femoral neck and pelvic fractures, intestinal obstruction and postoperative fistulas was significantly increased after preoperative radiotherapy in this group of patients [24]. Thus, a careful selection to choose the appropriate patients for neoadjuvant therapy is mandatory. In our series, older patients received preoperative treatment less frequently compared to younger patients.

Few limitations of the present study need to be addressed. The study was designed retrospectively, thus a selection bias cannot be ruled out. However, the active policy in our institution is to offer surgical treatment to the vast majority of older people who are referred to our clinic. Certainly, it is possible that few patients with rectal cancer were not seen by surgeons, as they were considered to be too frail to undergo surgery primarily, thus a conservative approach was chosen instead.

## Conclusions

Rectal cancer resection can be performed safely in selected older patients. Age itself should not be considered as a risk factor for postoperative complications. Attention should be paid to neurological and cardiovascular diseases, as they may increase the risk for an eventful postoperative course.

## Abbreviations

TEM: Transanal endoscopic microsurgery
UICC: Union internationale contre le cancer.
